# Supplementary material for: Plasmodium ARK2 and EB1 drive unconventional spindle dynamics, during chromosome segregation in sexual transmission stages
Source: Nat Commun. 2023 Sep 13;14:5652. doi: 10.1038/s41467-023-41395-3 (PMC10499817; doi:10.1038/s41467-023-41395-3)
Supplement: Supplementary file 1 — Supplementary Information File [file 41467_2023_41395_MOESM1_ESM.pdf]

Supplementary Information

***Plasmodium* ARK2 and EB1 drive unconventional spindle dynamics, during chromosome segregation in sexual transmission stages**

Mohammad Zeeshan[1], Edward Rea[1], Steven Abel[2], Kruno Vukušić[3], Robert Markus[1], Declan Brady[1], Antonius Eze[1#], Ravish Rashpa[4], Aurelia C. Balestra[4], Andrew R. Bottrill[5], Mathieu Brochet[4], David S. Guttery[6], Iva M. Tolić[3], Anthony A. Holder[7], Karine G. Le Roch[2], Eelco C. Tromer[8], Rita Tewari[1]*

1. Supplementary notes 1 and 2
2. Supplementary Figures 1-12 and figure legends
3. Uncropped blots (Supplementary fig. 6C)

Supplementary notes

Note1

Comparative genomics and subcellular location of AK paralogues suggest that Plasmodium ARK2 is a spindle-based AK

To gain insight into the roles of *P. berghei* ARKs in cell division, we first re-evaluated their evolutionary history (**Fig. 1, Supplementary Fig. 1)**. We updated phyletic profiles [1-3] for AKs, related mitotic kinases and their location-specific scaffolds and activators, using a selection of eukaryotes (**Fig. 1B**, **Supplementary data 1**). We found a pervasive loss of Aurora scaffold proteins (Survivin, Borealin, TPX2, Cep192 and BORA) in the common ancestor of Plasmodium and Toxoplasma, correlating with the loss of the widely conserved centromere/spindle kinases (Mps1, Bub1 and Polo), and the expansion of both CPC subunit INCENP (2 paralogues) and the AK family (3 paralogues). Flowering plants (*Arabidopsis thaliana*) and kinetoplastids (*Trypanosoma brucei*, Tb) have similar losses to a different extent. Kinetoplastids provide the only known example of functional replacement of Survivin and Borealin by TbCPC2 [4] and of Cep192 by the basal body scaffold TbABP67 [5] (**Fig. 1B, Supplementary data 1**). To explore whether common patterns of AK sub-functionalization after duplication might apply to ARK1-2-3, we used the five different subcellular locations of AK as described for scaffolds/activators of AKs in model eukaryotes: (I) centromere, (II) spindle MT, (III) spindle pole, (IV) central spindle, and (V) cyto/nucleoplasm (**Fig. 1A**). The reported subcellular location of each paralogue was reviewed to determine whether a shift in location occurred after the inferred duplication point [6]. This analysis corroborated the proposed pattern of recurrent sub-functionalization following the first duplication event into ‘equatorial’ (CPC-associated) and ‘polar’ (spindle-associated) AK paralogues (**Supplementary Fig. 1)**. All *Plasmodium* spp. and *T. gondii* duplications are shared: each paralogue being one-to-one orthologous (Reininger et al., 2011), and strongly suggesting a similar function. TgARK1 is located at the centromere and associated with INCENP1 and 2, but not at the central spindle or cleavage furrow during cytokinesis, unlike TgARK3 [7]. PfARK1 is located at or near the spindle pole/MTOC [8] but lower resolution imaging precluded more exact positioning, e.g. spindle pole or centromere. This suggests that in the common ancestor of Hematozoa (i.e. *Plasmodium spp*) and Coccida (i.e. *T.* gondii) this may have been a

centromere-based equatorial-like AK. The second duplication event in mammals (Aurora B: Aurora C) and plants (AUR1:AUR2) produced paralogues with similar location profiles, targeting the equatorial AK in mammals, and the polar/spindle AK in plants. This distinction is less pronounced In kinetoplastids, with only one AK (AUR1) retaining ancestral function, and two other highly divergent paralogues (AUK2/3) [5]. TgARK2 and TgARK3 are associated with the spindle or spindle pole, consistent with sub-functionalization after duplication, although TgARK3 also appears to function at the cleavage furrow during cytokinesis. All apicomplexan ARK2 and ARK3 paralogues are considerably larger (~1500 to 3500 amino acid residues) than other AKs (~300 to 350 residues), including apicomplexan ARK1-like paralogues (**Supplementary Fig. 1)**. No structural features or clear conserved sequence were identified in Plasmodium ARK2/3, which may indicate regions binding to putative interaction partners (**Supplementary Fig. 1)**. In summary, Plasmodium ARK2 and ARK3 are highly divergent AK paralogues, but our evolutionary reconstructions strongly suggest a role for these proteins at the spindle and/or spindle pole.

Note 2

ARK2 is located in the nucleus throughout the *P. berghei* life cycle

To investigate the expression and subcellular location of ARK2, we generated a transgenic parasite line by single crossover recombination at the 3' end of the endogenous *ark2* locus to express a C-terminal GFP-tagged fusion protein **(Supplementary Fig. 2A)**. PCR analysis of genomic DNA using locus-specific diagnostic primers indicated correct integration of the GFP tagging construct **(Supplementary Fig. 2B).** ARK2-GFP parasites completed the full life cycle, with no detectable phenotype resulting from the GFP tagging. Expression and location of ARK2-GFP were assessed by live cell imaging; ARK2-GFP was observed in all developmental stages including asexual (blood schizogony and sporogony) **(Supplementary Fig. 2C, D)** and sexual (gametogony and ookinete development) (**Supplementary Fig. 2E, F**) stages. ARK2-GFP showed a punctate nuclear pattern with one or two focal points during blood schizogony (**Supplementary Fig. 2C**) and sporogony (**Supplementary Fig. 2D**). It was present at a single focal point with an additional more diffuse nuclear location during early stages of male gametogony (30 sec after activation) and in the zygote (2h after fertilization) (**Supplementary Fig. 2E,**

F), but in later stages it had a more dynamic location on the spindle and spindle pole as described in the results section. Interestingly, ARK2-GFP was not detected in mature asexual (merozoites and sporozoites) and sexual (male gametes and ookinetes) stages of development (**Supplementary Fig. 2C-F**).

Supplementary Fig. 1

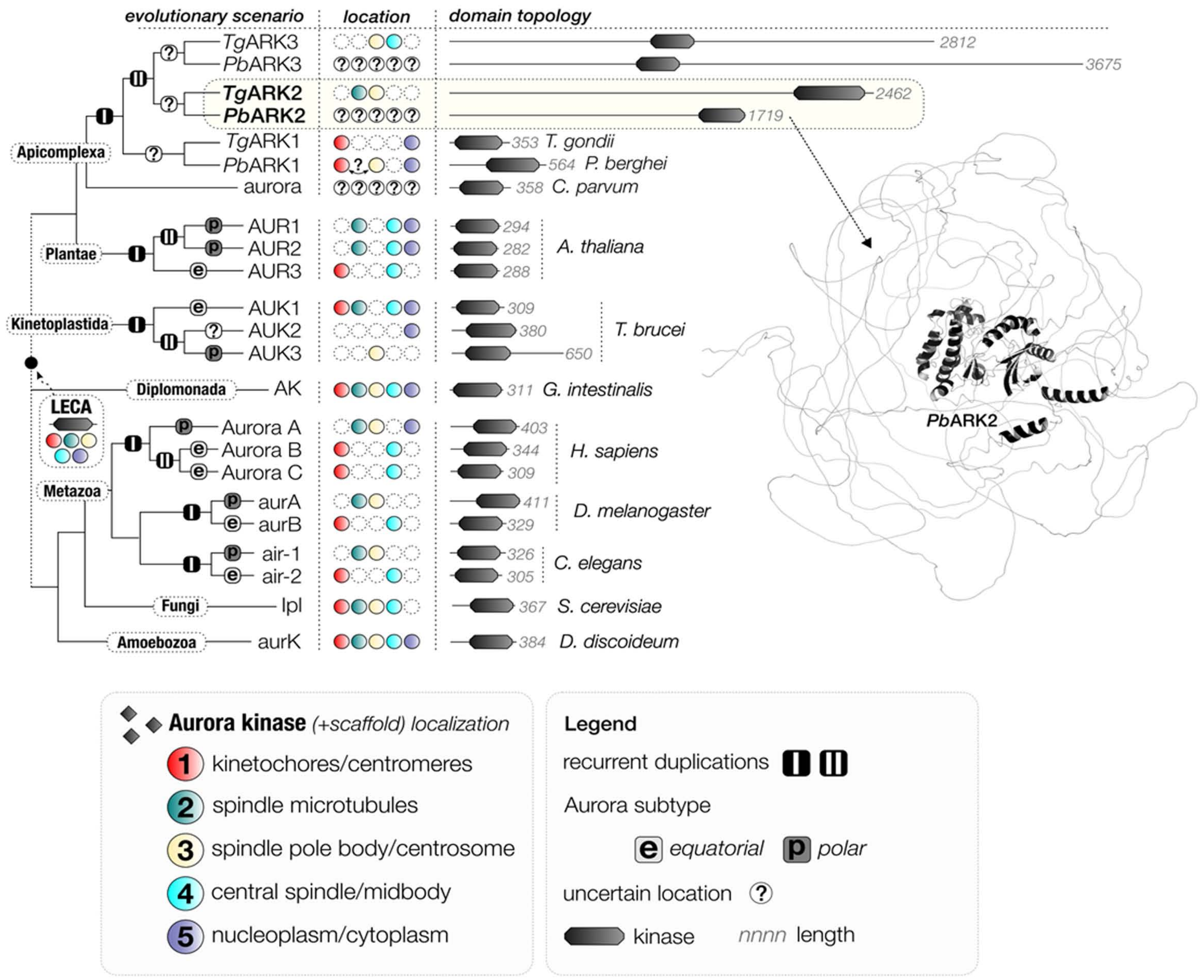

Supplementary Fig. 1. Comparative genomics of the Aurora kinase family reveal highly divergent paralogues among representatives of the apicomplexan lineages Hematozoa and Coccidia.

Recurrent duplication and sub-functionalisation in the AK family. Left: the phylogenetic relationships of Aurora paralogs. Light and dark grey boxes indicate the subtype: polar (p) or equatorial (e). I and II indicate points of recurrent duplication in the family. Middle: subcellular location of each Aurora paralog (if known); colours are the same as Figure 1A and shown below in the legend. Right: AK domain topology; note the extended length of ARK2 and ARK3 in Apicomplexa. Right bottom: Predicted structure of *P. berghei* ARK2 (predicted using ColabFold [AlphaFold2]).

Supplementary Fig. 2

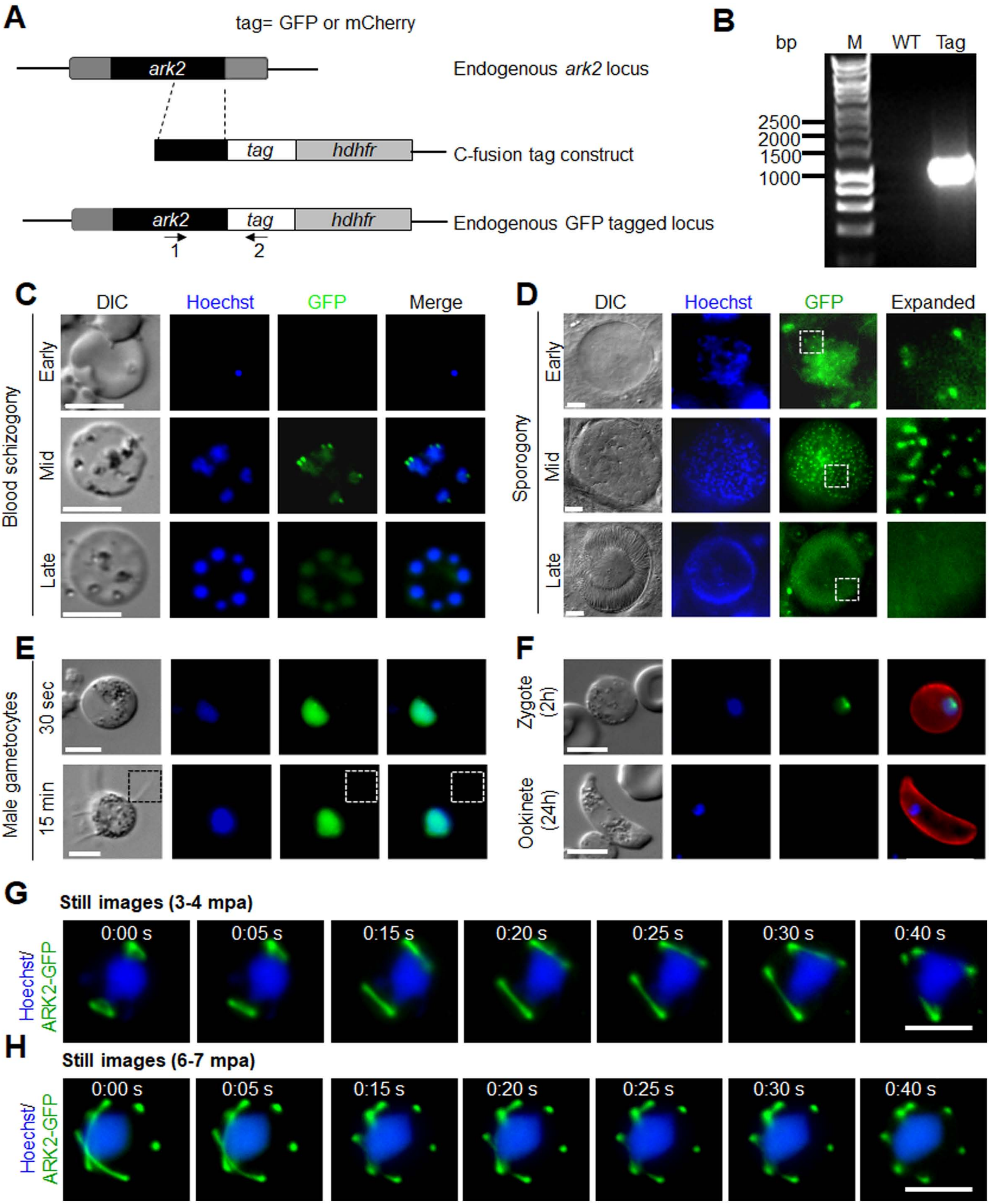

Supplementary Fig. 2. Generation of PbARK2-GFP/PbARK2-mCherry parasites and analysis of subcellular location of ARK2-GFP throughout the life cycle

(A) Schematic representation of the endogenous *Pbark2* locus, the GFP- and mCherry-tagging construct and the recombined *ark2* locus following single homologous recombination. Arrows 1 and 2 indicate the position of PCR primers used to confirm successful integration of the construct. **(B)** Diagnostic PCR of *ark2* and WT parasites using primers IntT204 (Arrow 1) and ol492 (Arrow 2). Integration of the ark2 tagging construct gives a band of 594 bp. Tag = ARK2-GFP parasite line. **(C)** Live cell imaging of ARK2-GFP parasites during erythrocytic schizogony showing one or two focal points of ARK2-GFP (green) per nucleus. DNA is stained with Hoechst dye (blue); scale bar = 5 µm. More than 50 images were analysed in more than 5 different experiments. **(D)** Live cell imaging of ARK2-GFP parasites during oocyst development in mosquitoes showing discrete foci of ARK2-GFP. DNA is stained with Hoechst dye (blue). More than 50 images were analysed in more than 5 different experiments. Scale bar = 5 µm. **(E)** Live cell imaging showing ARK2-GFP gametocytes at 30 sec and 15 min after activation. ARK2-GFP was not detected in free gametes (15 min gametocytes). DNA is stained with Hoechst dye (blue). More than 50 images were analysed in more than 5 different experiments. Scale bar = 5 µm. **(F)** Live-cell imaging showing ARK2-GFP location in zygote and ookinete. A cy3-conjugated antibody, 13.1, which recognises the protein P28 on the surface of zygotes and ookinetes was used to mark these stages (red). DNA is stained with Hoechst dye (blue). More than 50 images were analysed in more than 5 different experiments. Scale bar = 5 µm. **(G)** Still images (at every 5 s) showing dynamic location of ARK2-GFP in gametocytes within 3 to 4 min post activation (mpa) during male gametogony. DNA is stained with Hoechst dye (blue). More than 10 time lapses were analysed in more than 5 different experiments. Scale bar = 5 µm. **(H)** Still images (at every 5 s) showing dynamic location of ARK2-GFP within 6 to 7 mpa during male gametogony. DNA is stained with Hoechst dye (blue). More than 10-time lapses were analysed in more than 5 different experiments. Scale bar = 5 µm.

Supplementary Fig. 3

A

Time after activation	Localization/ phenotype	Number of gametocytes	Number of experiments	Representative images
30 sec-1 min	1 focal point	>85	>5	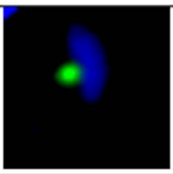
2-3 min	1 bridge/<br>2 focal point	>180	>5	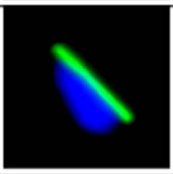 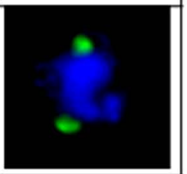
4-5 min	2 bridge/<br>4 focal point	>110	>5	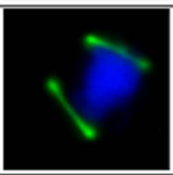 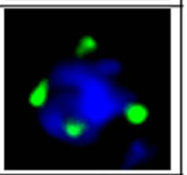
6-8 min	4 bridge/<br>8 focal point	>85	>5	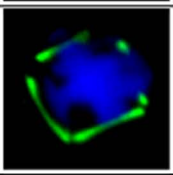 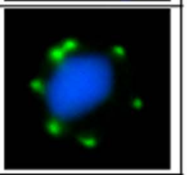
15-20 min	Diffused	>140	>5	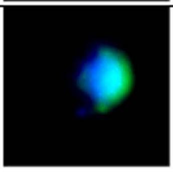

B

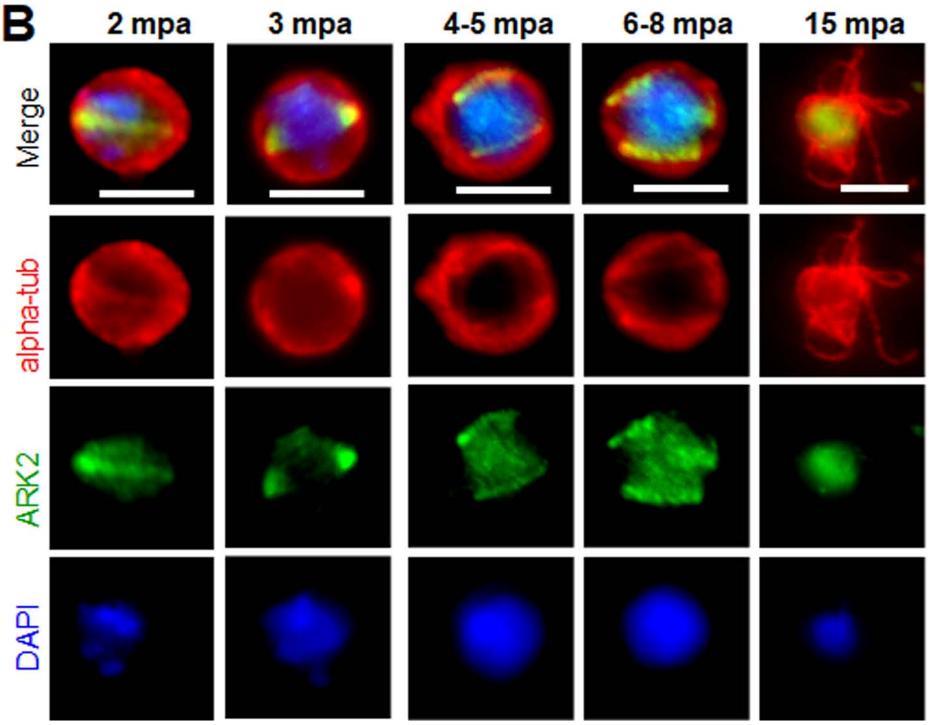

C

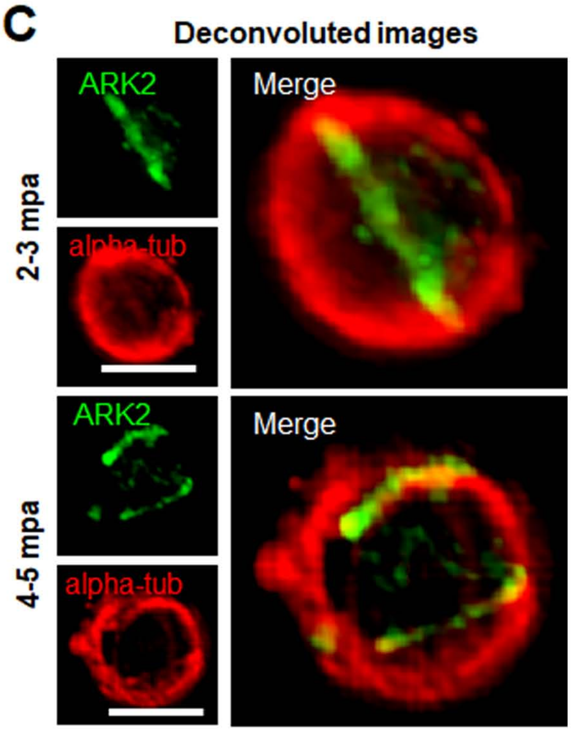

Supplementary Fig. 3. Quantification and location of ARK2 with tubulin antibody co-staining during male gametogony.

A. ARK2-GFP location at different time points following gametocytes activation. **(B)** More than 10 images were analysed in more than 2 different experiments. Immunofluorescence assay (IFA) showing location of ARK2 (green) and α-tubulin (red) in male gametocytes at different time points after activation**.** DNA is stained with DAPI (blue); mpa = min post activation; scale bar = 5 μm. **(C)** Deconvoluted images improve the resolution of ARK2 and show its colocation with spindle MTs. More than 10 images were analysed in more than 2 different experiments. Scale bar = 5 μm.

Supplementary Fig. 4

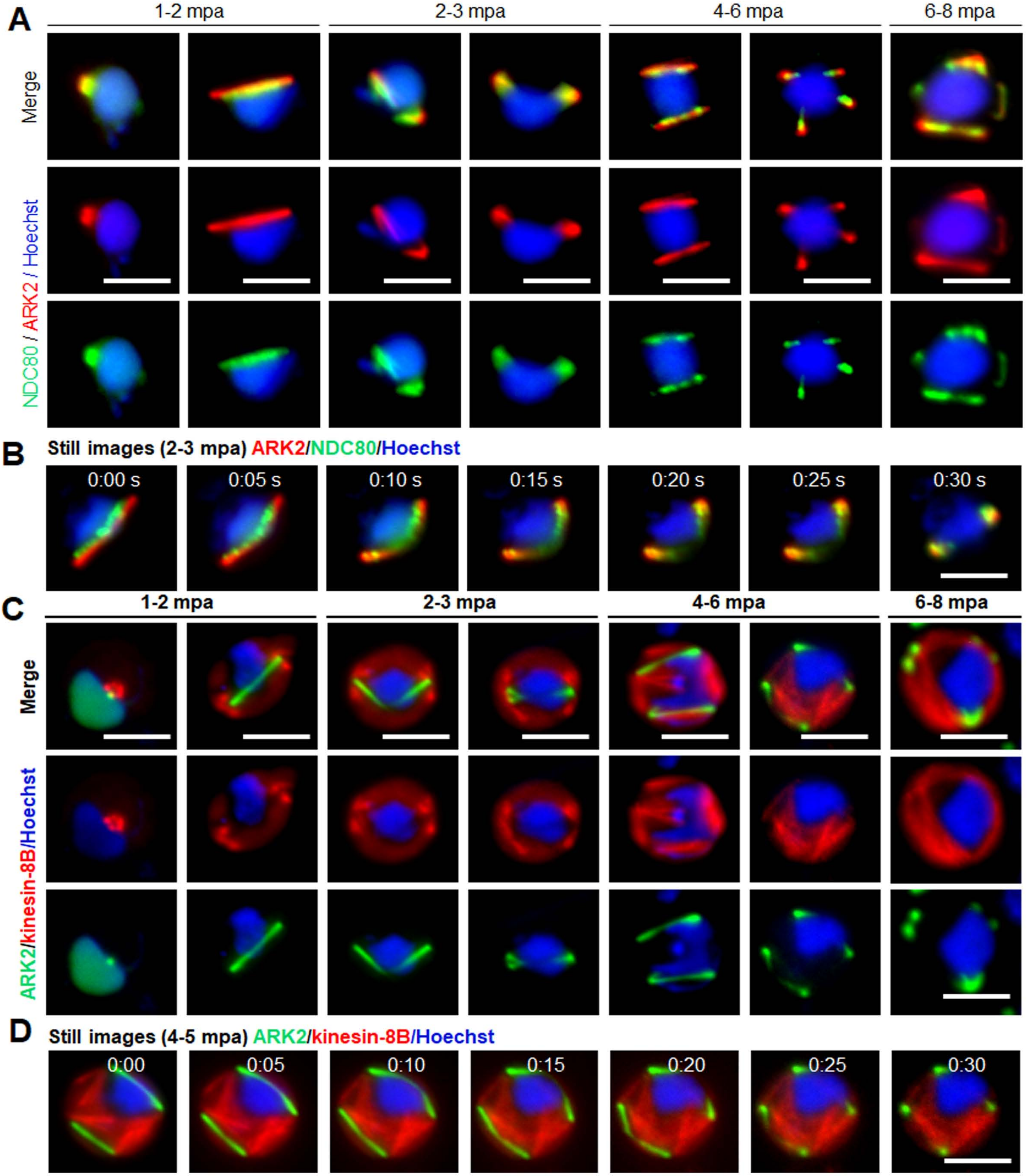

Supplementary Fig. 4. The location of ARK2 and various subcellular markers

(A) The location of ARK2-mCherry (red) and the kinetochore marker, NDC80-GFP (green) during male gametogony. DNA is stained with Hoechst dye (blue). More than 50 images were analysed in more than 5 different experiments. Scale bar = 5 µm**.** **(B)** Still images (at every 5 s) showing dynamic location of ARK2-mCherry and NDC80-GFP in gametocytes activated for 2 to 3 min. DNA is stained with Hoechst dye (blue). More than 10-time lapses were analysed in more than 5 different experiments. Scale bar = 5 µm. **(C)** The location of ARK2-GFP (green) and the basal body and axoneme marker, kinesin-8B-mCherry (red) during male gametogony. DNA is stained with Hoechst dye (blue). More than 50 images were analysed in more than 5 different experiments. Scale bar = 5 µm. **(D)** Still images (at every 5 s) showing dynamic location of ARK2-GFP and kinesin-8B-mCherry in gametocytes activated for 4 to 5 min. DNA is stained with Hoechst dye (blue). More than 10-time lapses were analysed in more than 5 different experiments. Scale bar = 5 µm.

Supplementary Fig. 5

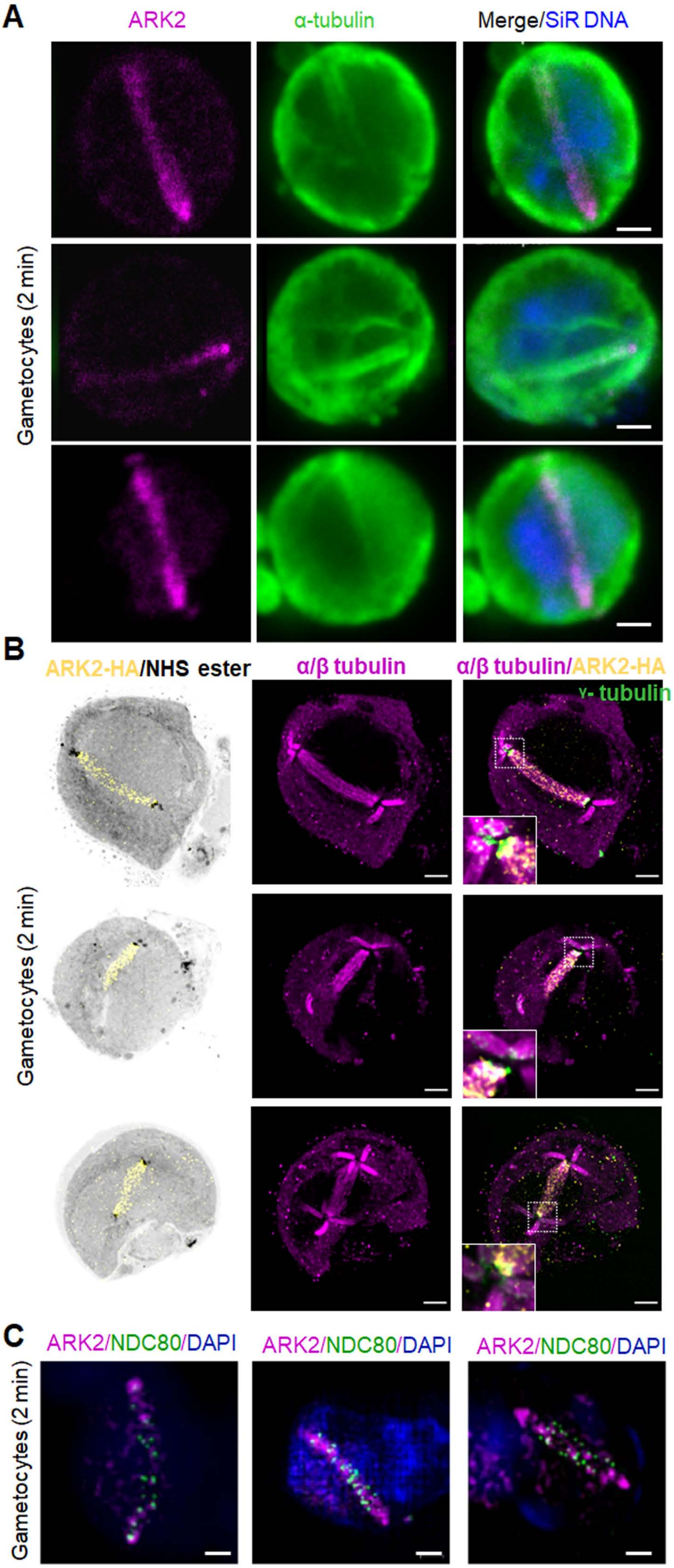

Supplementary Fig. 5. ARK2 associates with spindle MTs.

(A) STED confocal microscopy showing co-localization of ARK2 (purple) and α-tubulin (green) at spindle but not with cytoplasmic MTs in gametocytes activated for 2 min. DNA is stained with SiR DNA (blue). More than 10 images were analysed in more than 2 different experiments. Scale bar = 1 μm. **(B)** Expansion microscopy showing co-localization of ARK2 (yellow) and α/β tubulin (purple) staining at spindle but not at cytoplasmic MTs at 2 mpa. More than 10 images were analysed in more than 2 different experiments. Scale bar = 1 μm. **(C)** 3D-SIM image showing locations of ARK2 (purple) and NDC80 (green) at 2 mpa. DNA (blue) is stained with DAPI. More than 10 images were analysed in more than 2 different experiments. Scale bar = 1 μm.

Supplementary Fig. 6

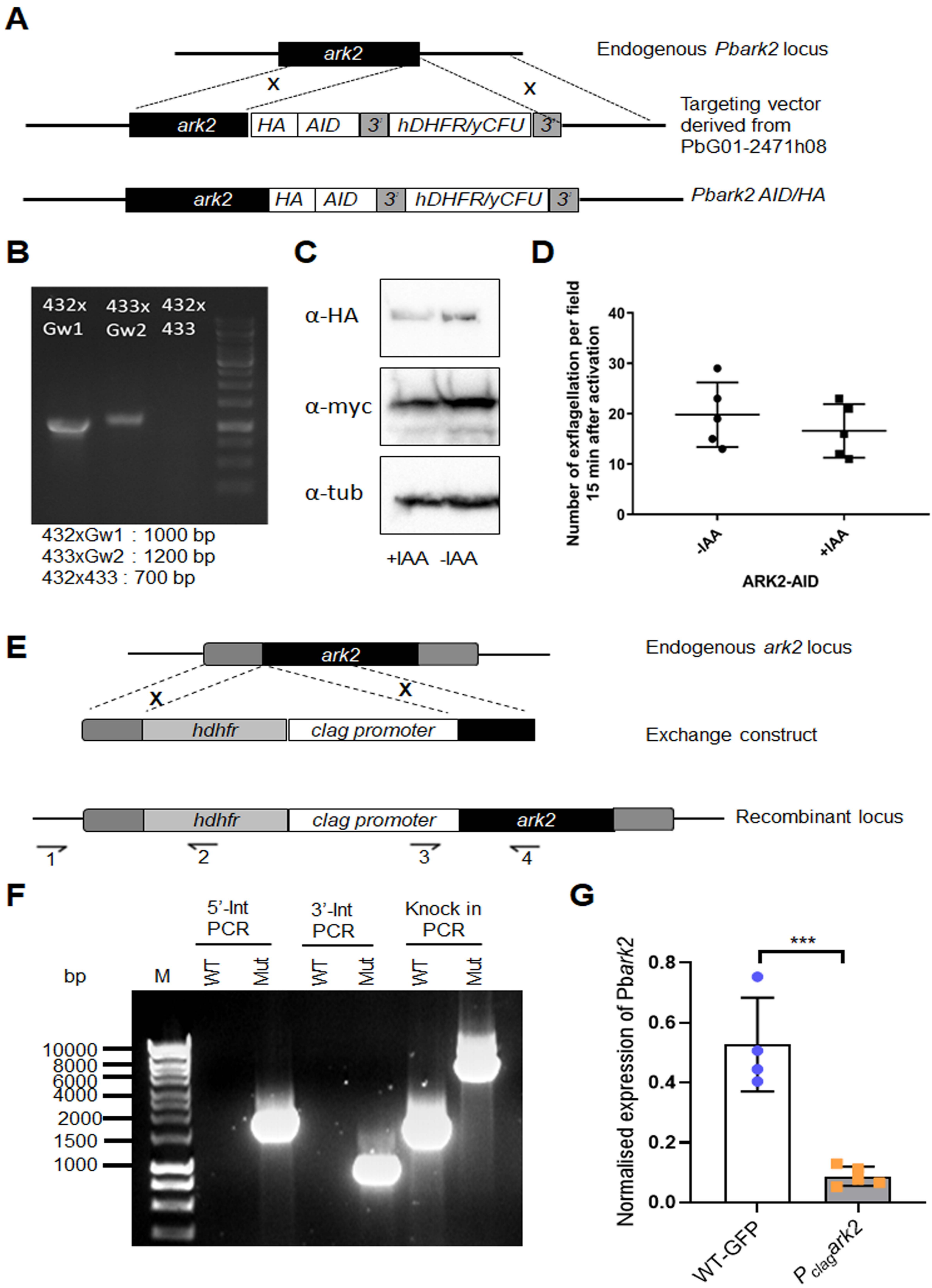

Supplementary Fig. 6. Generation and genotypic analysis of *Pb*ARK2-AID/HA and P_{clag}-*ark2* parasites.

(A) Schematic representation of auxin inducible degron (AID) strategy to generate *ARK2-AID/HA* parasites. (**B). I**ntegration PCR of the ARK2-AID/HA construct in the *ark2* locus. Oligonucleotides used for PCR genotyping are indicated, and agarose gels to analyse the corresponding PCR products from genotyping reactions are shown. Representative image of more than 3 experiments. (**C)** ARK2-AID/HA protein expression level as measured by western blotting upon addition of auxin to mature purified gametocytes; α-tubulin served as a loading control. **(D)** Male gametogony (Exflagellation rate) of ARK2-AID/HA as measured upon addition of auxin and without auxin to mature purified gametocytes. Shown is mean ± SEM; n = 5 independent experiments. **(E)** Schematic representation of the promoter swap strategy to construct *Pclag-ark2* parasites (placing ARK2 under the control of the clag promoter) by double homologous recombination. Arrows 1 and 2 indicate the primer positions used to confirm 5' integration and arrows 3 and 4 indicate the primers used to confirm 3' integration. **(F) I**ntegration PCR of the promotor swap construct into the *ARK2* locus. Primer 1 (IntPTD245) and primer 2 (5'-IntPTD) were used to confirm successful integration of the selectable marker, resulting in a band of 460 bp. Primer 3 (3'-intPTclag) and primer 4 (IntPTD243) were used to determine the successful integration of the clag promoter, resulting in a band of 571 bp. Primer 1 (IntPTD245) and primer 4 (IntPTD243) were used to confirm a complete knock-in of the construct with a band at 4.5 kb and the absence of a band at 2.1 kb. Representative image of more than 3 experiments. **(G)** qRT-PCR showing normalised expression of ARK2 transcripts in P_{clag}-*ark2* and WT-GFP parasites. Shown is mean ± SEM; n = 3 independent experiments. Student t test was used to examine significant difference. ***p<0.01.

Supplementary Fig. 7

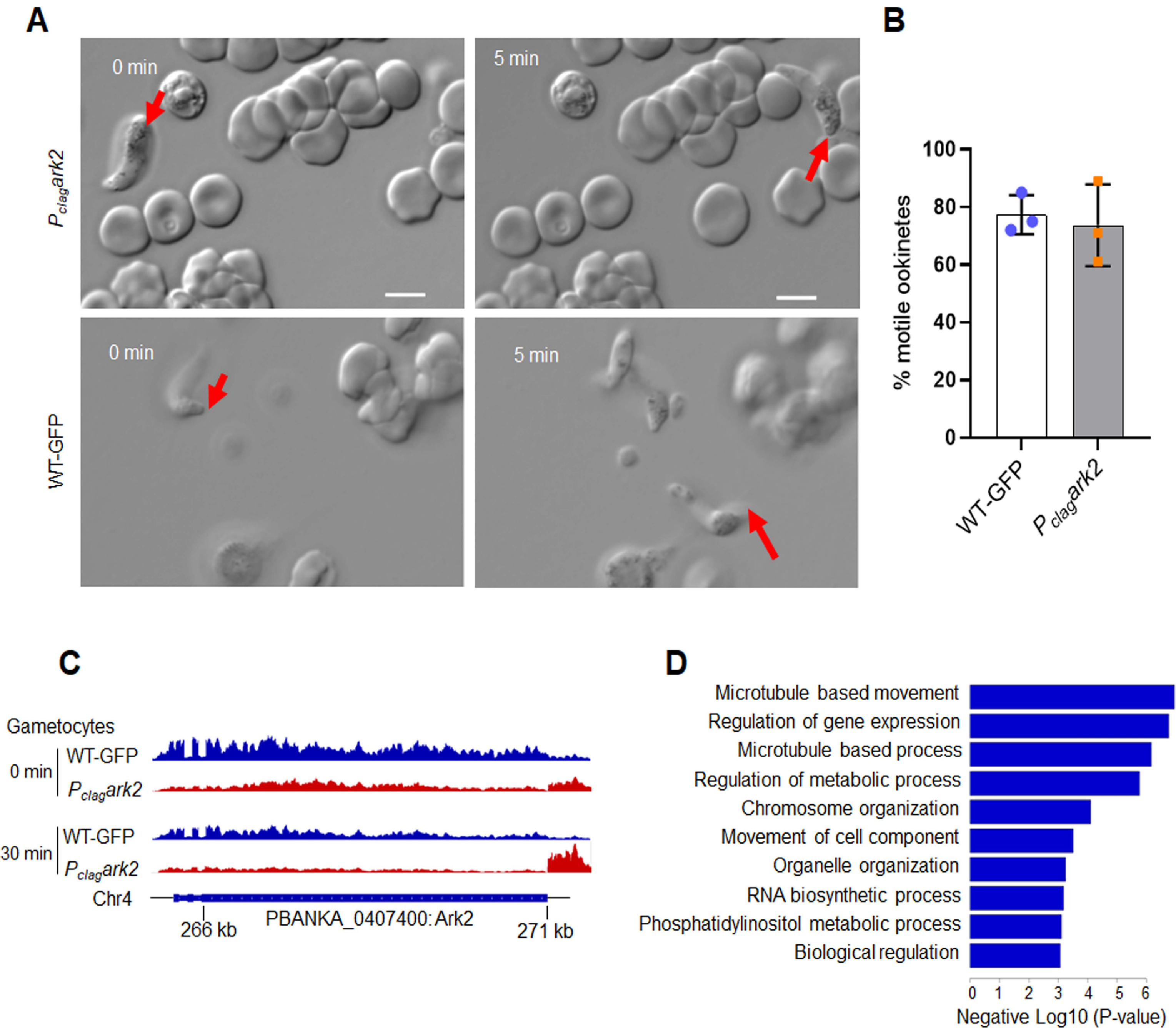

Supplementary Fig. 7. Analysis of ookinete motility of *P$_{clag}$-ark2* and WT-GFP parasites

(A) Representative frames from time-lapse videos of WT-GFP and *Pclag-ark2* ookinetes in matrigel. Red arrow indicates the apical end of the ookinetes. Bar = 5 µm. More than 5-time lapse were recorded in at least two experiments. **(B)** Graph shows a comparison of WT-GFP and *Pclag-ark2* ookinete motility. (Error bar ± SD; n=3 independent experiments; >20 ookinetes were analysed for each experiment). **(C)** RNA sequence analysis showing downregulated transcript of ARK2 in *Pclag-ark2* parasites. **(D)** Gene ontology enrichment analysis showing the most affected genes involved in various biological processes.

Supplementary Fig. 8

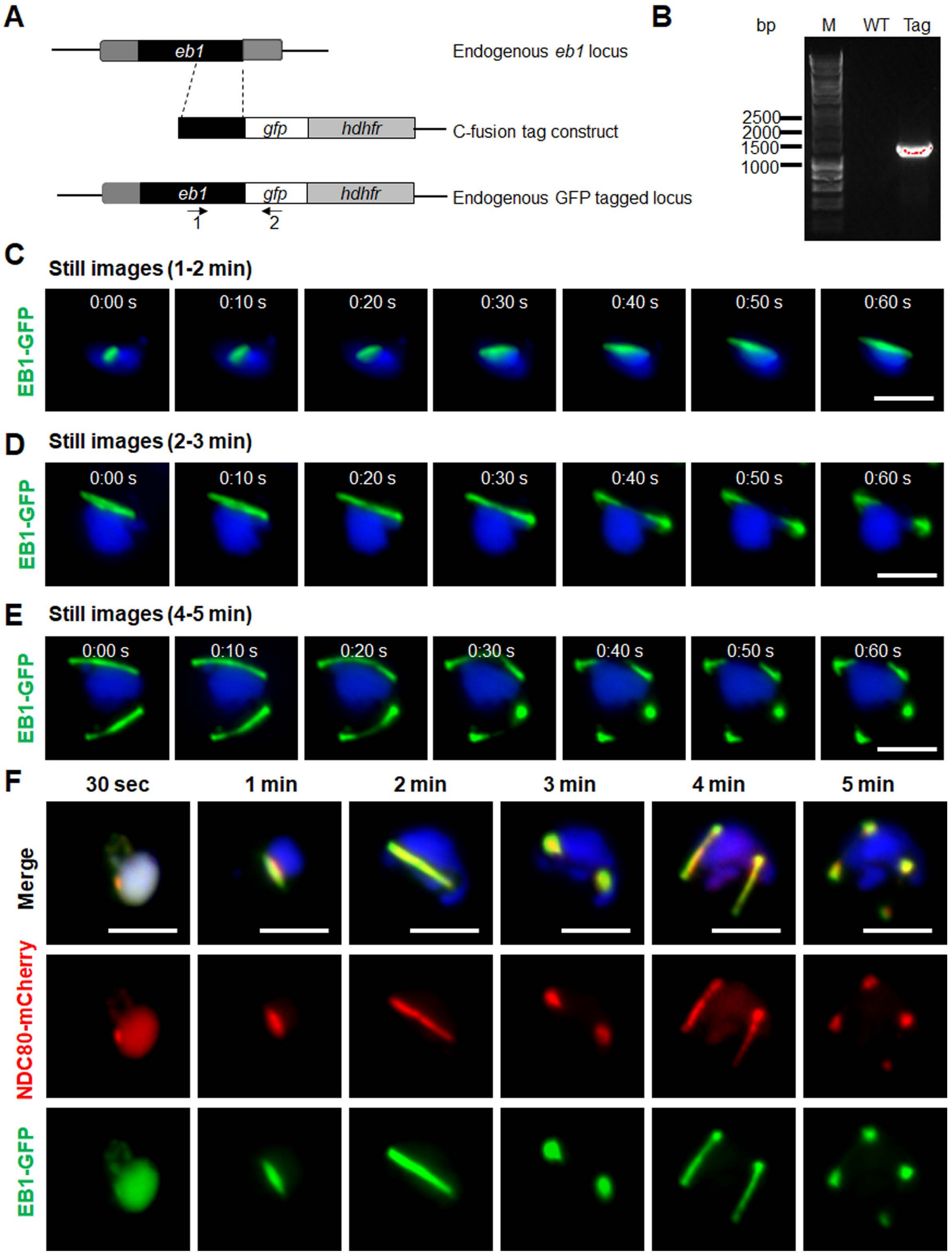

Supplementary Fig. 8. Generation of PbEB1-GFP parasites and analysis of PbEB1-GFP location during gametogony

(A) Schematic representation of the endogenous *Pbeb1* locus, the GFP-tagging construct and the recombined *eb1* locus following single homologous recombination. Arrows 1 and 2 indicate the position of PCR primers used to confirm successful integration of the construct. **(B)** Diagnostic PCR of *eb1* and WT parasites using primers IntT264 (Arrow 1) and ol492 (Arrow 2). Integration of the EB1 tagging construct gives a band of 1267 bp. Tag = EB1-GFP parasite line. Representative image of more than 3 experiments. **(C)** Still images (at every 5 s) showing dynamic location of EB1-GFP in activated gametocytes at 1-2 min during male gametogony. DNA is stained with Hoechst dye (blue). More than 10-time lapses were analysed in more than 5 different experiments. Scale bar = 5 µm. **(D)** Still images (at every 5 s) showing dynamic location of EB1-GFP in activated gametocytes at 2 to 3 mpa. DNA is stained with Hoechst dye (blue). More than 10-time lapses were analysed in more than 5 different experiments. Scale bar = 5 µm. **(E)** Still images (at every 5 s) showing dynamic location of EB1-GFP in activated gametocytes at 4 to 6 mpa. DNA is stained with Hoechst dye (blue). More than 10-time lapses were analysed in more than 5 different experiments. Scale bar = 5 µm. **(F)** The location of EB1-GFP (green) and the kinetochore marker, NDC80-mCherry (red) during male gametogony. DNA is stained with Hoechst dye (blue). More than 50 images were analysed in more than 3 different experiments. Scale bar = 5 µm.

Supplementary Fig. 9

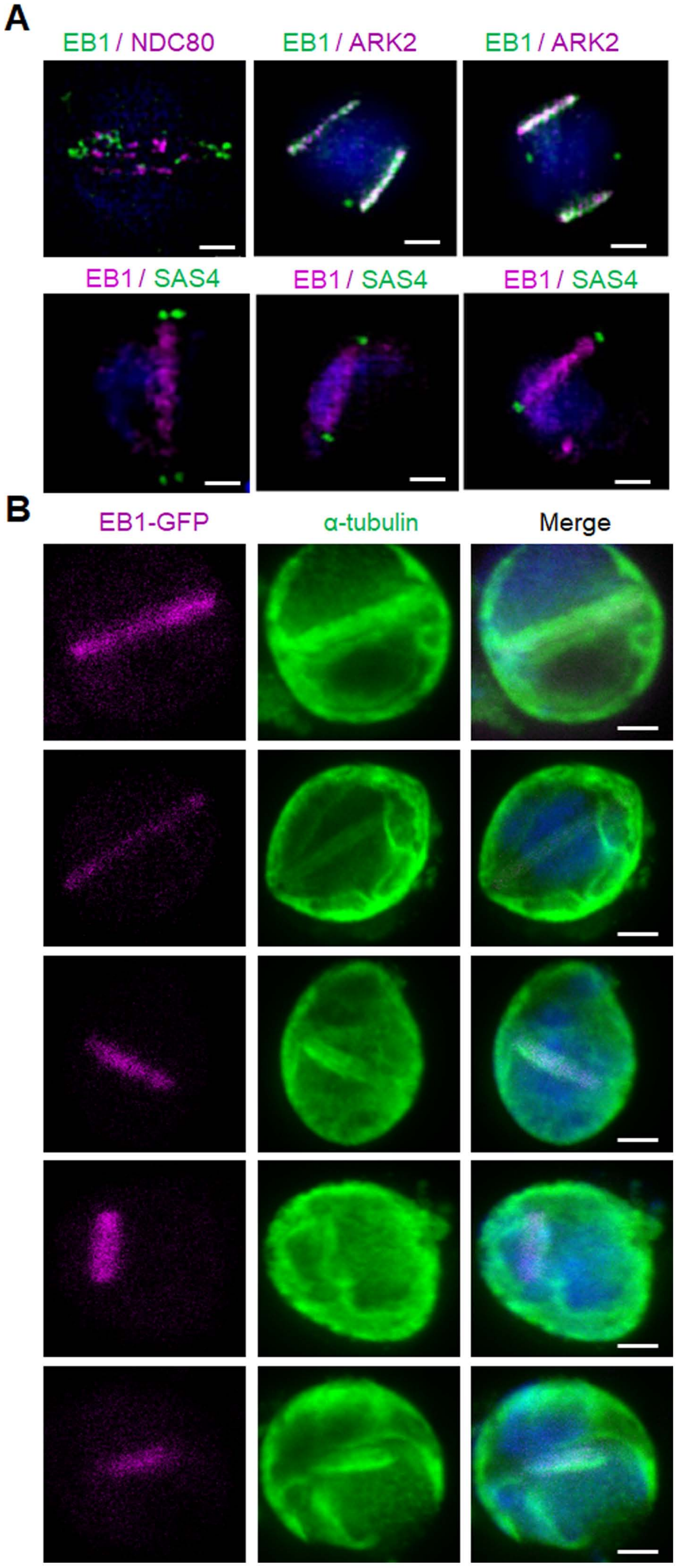

Supplementary Fig. 9. EB1 associates with spindle MTs.

(A) 3D-SIM image showing location of EB1 (green) with NDC80 (purple) in gametocyte activated for 2 min; and EB1 (green) with ARK2 (purple) in gametocytes activated for 4 min. 3D-SIM images showing location of EB1 (purple) and cytoplasmic SAS4 (green) in gametocyte activated for 2 min. DNA is stained with Hoechst dye (blue). More than 10 images were analysed in more than 2 different experiments. Scale bar = 1 μm. (B) STED confocal microscopy showing co-localization of EB1 (purple) and α-tubulin (green) at spindle but not with cytoplasmic MTs in gametocytes activated for 2 min. DNA is stained with SiR DNA (blue). More than 10 images were analysed in more than 2 different experiments. Scale bar = 1 μm.

Supplementary Fig. 10

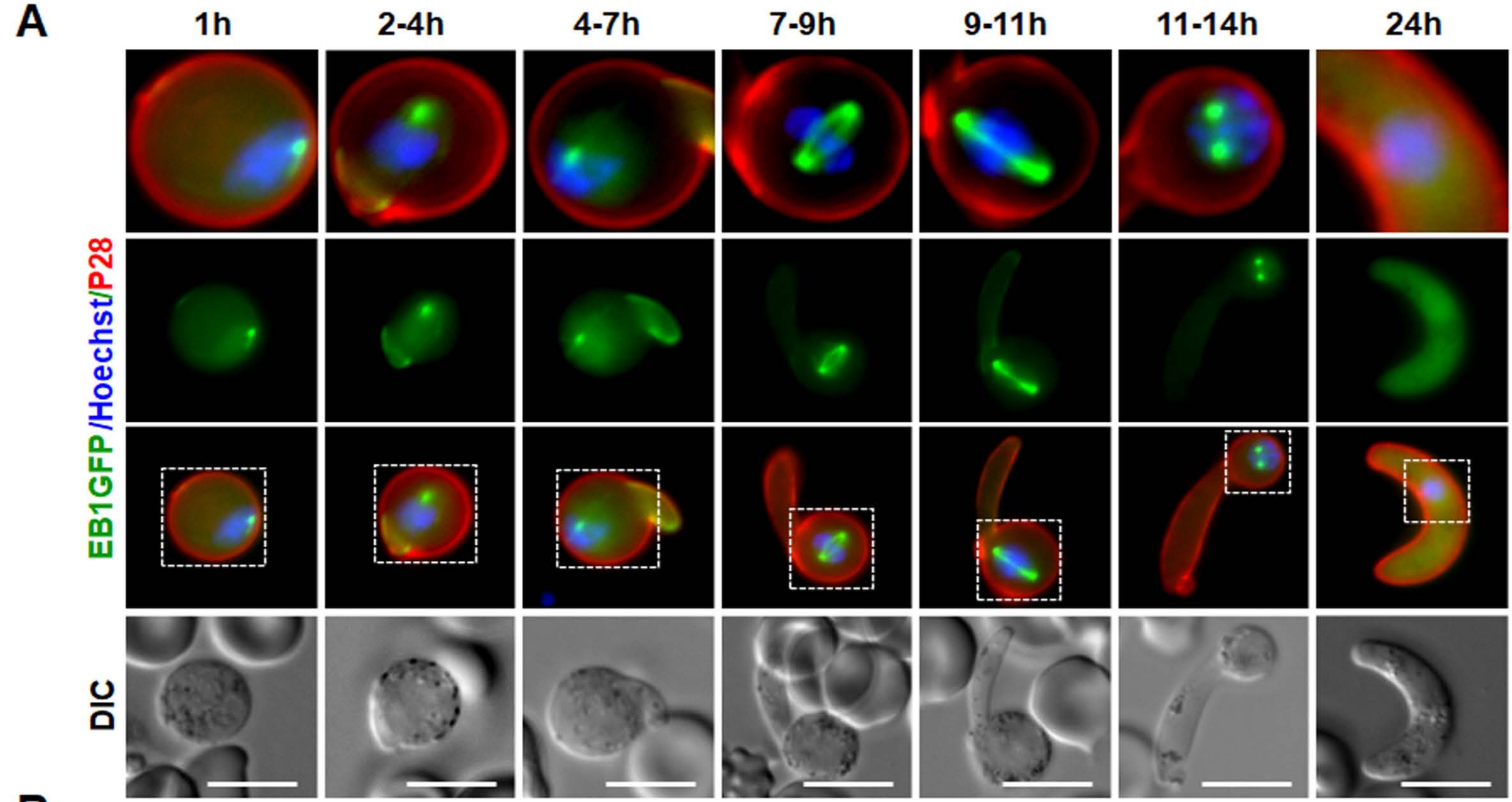

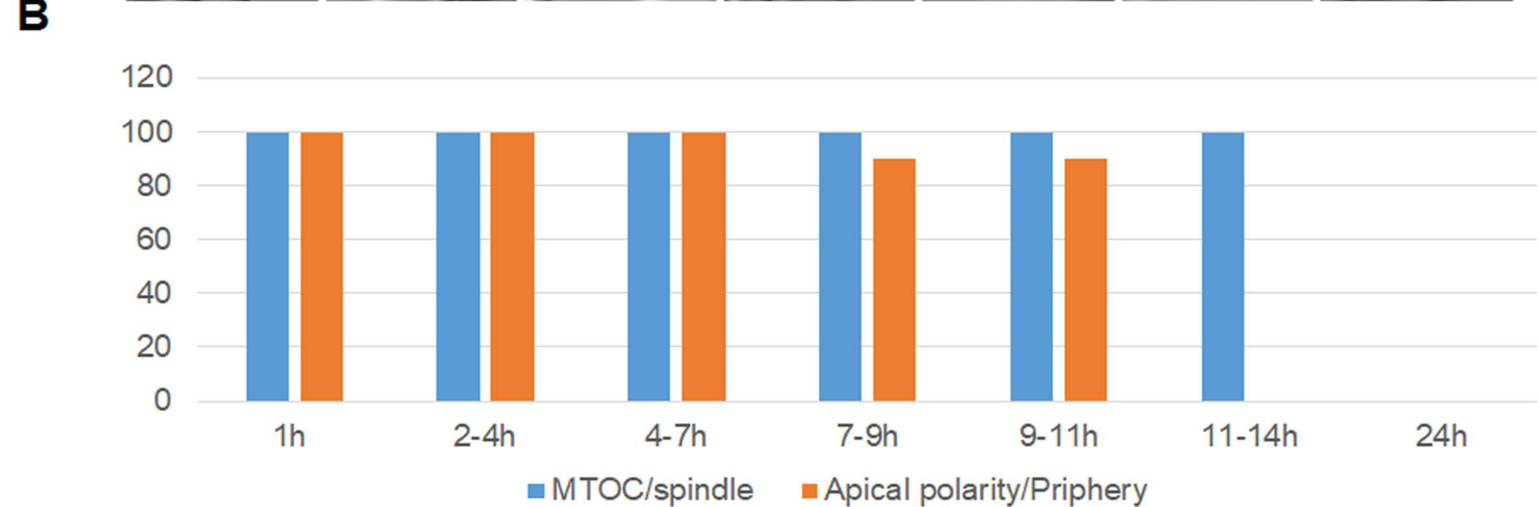

Supplementary Fig. 10. PbEB1-GFP is located at the apical end of the parasite and at the putative MTOC and spindle like PbARK2-GFP during ookinete development

(A) Live-cell imaging shows that EB1-GFP is located at the MT organising centre (MTOC) and spindles in the nucleus during ookinete development and then disappears in mature ookinetes (24 h). It is also located at the apical end of the growing protuberance during zygote to ookinete transition. A cy3-conjugated antibody, 13.1, which recognises the protein P28 on the surface of zygotes and ookinetes was used to mark these stages (red). More than 10 images were analysed in more than 3 different experiments. Scale bar = 5 μm. **(B)** Bar diagram shows the prevalence of EB1-GFP distribution at MTOC/spindles and apical pole/periphery during ookinete development.

Supplementary Fig. 11

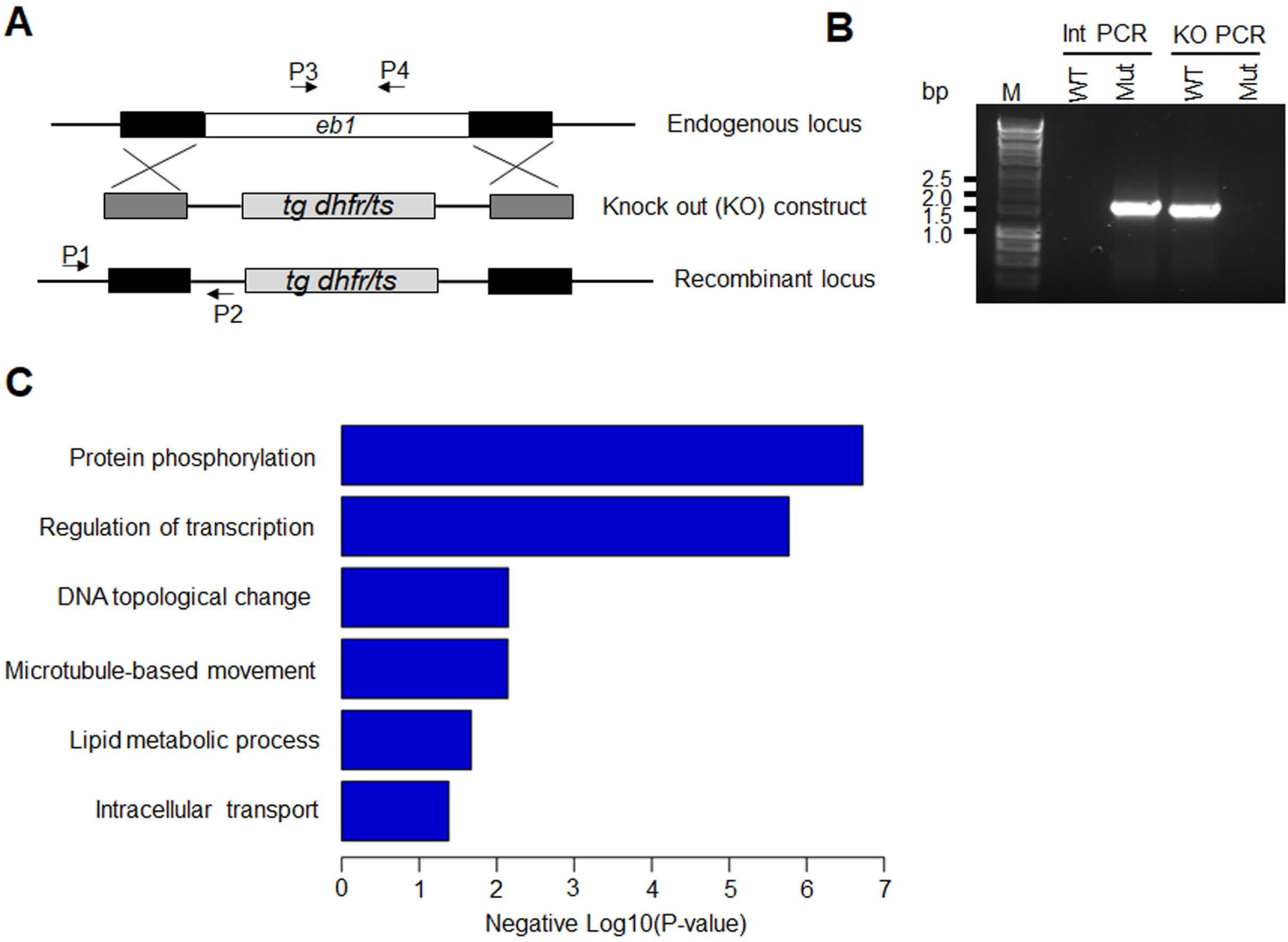

Supplementary Fig. 11. Generation and genotypic analysis of *Δeb1* parasites

(A) Schematic representation of the endogenous *eb1* locus, the targeting knockout construct and the recombined *eb1* locus following double homologous crossover recombination. Arrows 1 and 2 indicate PCR primers used to confirm successful integration in the *eb1* locus following recombination, and arrows 3 and 4 indicate PCR primers used to show deletion of the *eb1* gene. (**B)** Integration PCR of the *eb1* locus in WTGFP (WT) and knockout (Mut) parasites using primers: integration primer and ol248. Integration of the targeting construct gives band of expected size. Representative image of more than 3 experiments. **(C)** Gene ontology enrichment of upregulated genes in global transcriptomic analysis of *Δeb1* gametocytes activated for 30 min, showing where the most affected genes are involved in various biological processes.

Supplementary Fig. 12

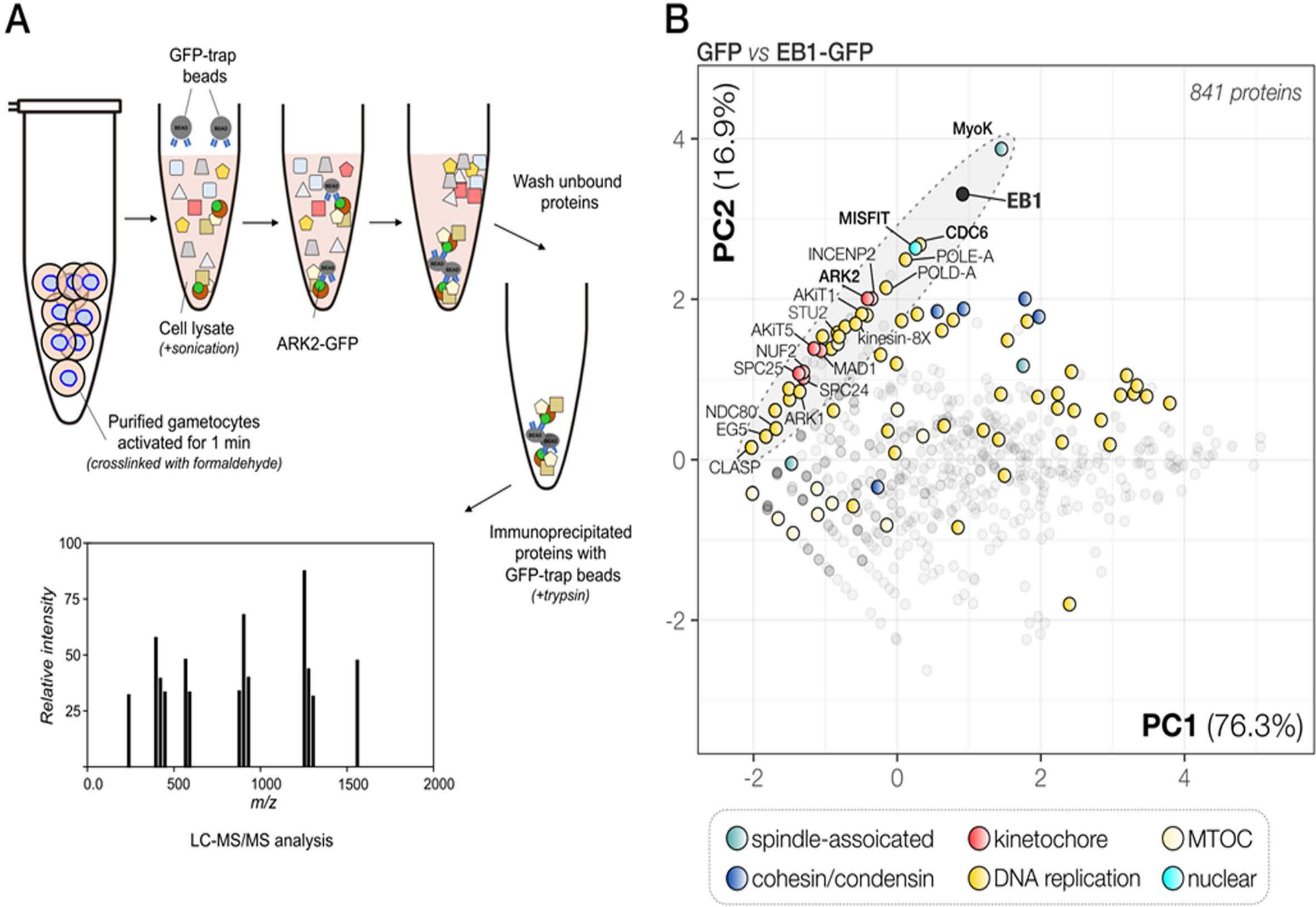

Supplementary Fig. 12. Pulldown workflow and principal component analysis of EB1-GFP IP LC-MS/MS

(**A**) Workflow for immunoprecipitation experiment using GFP-trap beads and gametocyte crosslinked lysates, trypsin digestion and mass spectrometry analysis to identify GFP-tagged protein interaction partners. (**B**) Projection of the first two components of a principal component analysis (PCA) of unique peptides identified by mass spectrometry from EB1-GFP or GFP-alone control immunoprecipitates using the GFP-trap system (raw data: **Supplementary data 3**). A subset of proteins is highlighted on the map based on relevant functional categories.

Uncropped blots (Supplementary fig 6C): Box shows the represented blot.

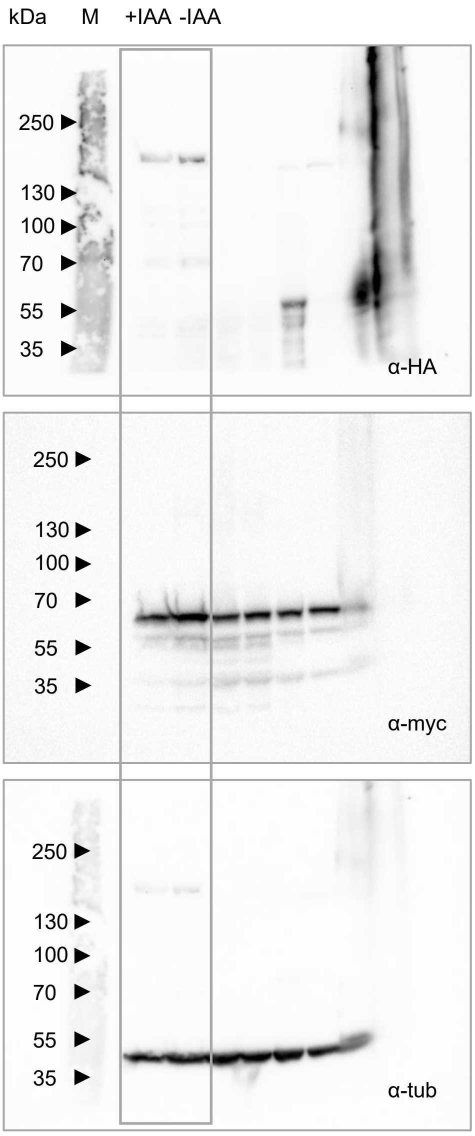

Supplementary References

1 van Hooff, J. J., Tromer, E., van Wijk, L. M., Snel, B. & Kops, G. J. Evolutionary dynamics of the kinetochore network in eukaryotes as revealed by comparative genomics. *EMBO Rep* **18**, 1559-1571, doi:10.15252/embr.201744102 (2017).

2 Komaki, S. *et al.* Molecular convergence by differential domain acquisition is a hallmark of chromosomal passenger complex evolution. *Proc Natl Acad Sci U S A* **119**, e2200108119, doi:10.1073/pnas.2200108119 (2022).

3 Kops, G., Snel, B. & Tromer, E. C. Evolutionary Dynamics of the Spindle Assembly Checkpoint in Eukaryotes. *Curr Biol* **30**, R589-R602, doi:10.1016/j.cub.2020.02.021 (2020).

4 Davids, B. J., Williams, S., Lauwaet, T., Palanca, T. & Gillin, F. D. Giardia lamblia aurora kinase: a regulator of mitosis in a binucleate parasite. *Int J Parasitol* **38**, 353-369, doi:10.1016/j.ijpara.2007.08.012 (2008).

5 Akiyoshi, B. Analysis of a Mad2 homolog in Trypanosoma brucei provides possible hints on the origin of the spindle checkpoint. *bioRxiv*, doi:https://doi.org/10.1101/2020.12.29.424754 (2020).

6 Hochegger, H., Hegarat, N. & Pereira-Leal, J. B. Aurora at the pole and equator: overlapping functions of Aurora kinases in the mitotic spindle. *Open Biol* **3**, 120185, doi:10.1098/rsob.120185 (2013).

7 Berry, L. *et al.* The conserved apicomplexan Aurora kinase TgArk3 is involved in endodyogeny, duplication rate and parasite virulence. *Cell Microbiol* **18**, 1106-1120, doi:10.1111/cmi.12571 (2016).

8 Reininger, L., Wilkes, J. M., Bourgade, H., Miranda-Saavedra, D. & Doerig, C. An essential Aurora-related kinase transiently associates with spindle pole bodies during Plasmodium falciparum erythrocytic schizogony. *Mol Microbiol* **79**, 205-221, doi:10.1111/j.1365-2958.2010.07442.x (2011).
