# Supplementary material for: Plasmodium ARK2 and EB1 drive unconventional spindle dynamics, during chromosome segregation in sexual transmission stages
Source: Nat Commun. 2023 Sep 13;14:5652. doi: 10.1038/s41467-023-41395-3 (PMC10499817; doi:10.1038/s41467-023-41395-3)
Supplement: Supplementary file 3 — Description of Additional Supplementary Files [file 41467_2023_41395_MOESM3_ESM.docx]

***Plasmodium* ARK2 and EB1 drive unconventional spindle dynamics, during chromosome segregation in sexual transmission stages**

 Mohammad Zeeshan^1^, Edward Rea^1^, Steven Abel^2^, Kruno Vukušić^3^, Robert Markus^1^, Declan Brady^1^_,_ Antonius Eze^1#^, Ravish Rashpa^4^, Aurelia C. Balestra^4^, Andrew R. Bottrill^5^, Mathieu Brochet^4^, David S. Guttery^6^, Iva M. Tolić^3^, Anthony A. Holder^7^, Karine G. Le Roch^2^, Eelco C. Tromer^8^, Rita Tewari^1^*****

**Supplementary data**

## **Supplementary data 1.** Overview of genomes and sequences used for generating Figure 1B.

### Supplementary data 2. List of genes differentially expressed between *P_clag-_ark2* and WT-GFP gametocytes.

### RNA sequencing analysis describing the expression pattern of various genes in *P_clag-_ark2* gametocytes in comparison to WT-GFP gametocytes activated for 30 min.

**Supplementary data 3**. List of proteins and unique peptides values for GFP-trap immunoprecipitants.

Spreadsheet (excel) file with unique peptide values for GFP-trap immunoprecipitant for gametocytes 1 minute after activation for WT-GFP, ARK2-GFP and EB1-GFP parasites. NAs are set to zero (0). Specific protein groups that belong to a similar functional class (e.g. replication machinery, kinetochore etc) are colour coded according to the scheme visualised in Fig 5A and Fig 8A. Five parts of the table are present: (1) gene details; containing gene name, manual annotations, amino acid number (AA) and molecular weight (MW), (2) correlations; Pearson (p) and Spearman (s, rank) correlation values for ARK2 and EB1, (3) PCA, principal components, (4) % coverage of the proteins by peptides identified in MS/MS, (5) unique peptide values; NA is -, and * indicates that single peptide calls are to be approached with suspicion (minimal of 2 is usual cut-off), (6) gene details II; for GO terms and OG definitions that can be found at PlasmoDB (https://plasmodb.org/).

**Supplementary data 4**. List of genes differentially expressed between *Δeb1* and WT-GFP gametocytes activated for 30 min

**Supplementary data 5.**Oligonucleotides used in this study.

**Supplementary Movies**

**Supplementary Movie 1**. Time lapse video showing ARK2-GFP focal point extending to form a bridge-like spindle and breaking into two halves in gametocytes 1 to 2 min after activation. Still images used in Fig 2B.

**Supplementary Movie 2**. Time lapse video showing two ARK2-GFP bridge-like spindles breaking and producing four focal points in gametocytes 3 to 4 min after activation. Still images used in Fig S1G.

**Supplementary Movie 3**. Time lapse video showing four ARK2-GFP bridge-like spindles breaking and producing eight focal points in gametocytes 6 to 8 min after activation. Still images used in Fig S1H.

**Supplementary Movie 4**. Time lapse video showing ARK2-mCherry and NDC80-GFP dynamics in gametocytes activated for 1 to 2 min. Still images used in Fig 2D.

**Supplementary Movie 5**. Time lapse video showing ARK2-mCherry and NDC80-GFP dynamics in gametocytes activated for 2 to 3 min. Still images used in Fig S2D.

**Supplementary Movie 6**. Time lapse video showing ARK2-GFP and kinesin-8B-mCherry dynamics in activated gametocytes for 2-3 min. Still images used in Fig 2F.

**Supplementary Movie 7**. Time lapse video showing ARK2-GFP and kinesin-8B-mCherry dynamics in gametocytes activated for 4 to 6 min. Still images used in Fig S2F.

**Supplementary Movie 8**. Gliding motility of *Pclag-ark2* ookinetes. Still images used in Fig S4A

**Supplementary Movie 9**. **Gliding motility of *WT-GFP* ookinetes. Still images used in Fig S4A**

**Supplementary Movie 10**. Time lapse video showing EB1-GFP focal point extending to form a bridge like spindle in activated gametocytes for 1-2 min. Still images used in Fig S5C.

**Supplementary Movie 11**. Time lapse video showing EB1-GFP bridge breaking into two halves and accumulating at two focal points in a gametocyte activated for 2 to 3 min. Still images used in Fig S5D.

**Supplementary Movie 12**. Time lapse video showing two bridges of EB1-GFP breaking into four halves and accumulating at four focal points in a gametocyte activated for 2 to 3 min. Still images used in Fig S5E.
