# Supplementary material for: Plasmodium ARK2 and EB1 drive unconventional spindle dynamics, during chromosome segregation in sexual transmission stages
Source: Nat Commun. 2023 Sep 13;14:5652. doi: 10.1038/s41467-023-41395-3 (PMC10499817; doi:10.1038/s41467-023-41395-3)
Supplement: Supplementary file 21 — Reporting Summary [file 41467_2023_41395_MOESM21_ESM.pdf]

nature portfolio

Corresponding author(s): Rita Tewari

Last updated by author(s): Aug 11, 2023

Reporting Summary

Nature Portfolio wishes to improve the reproducibility of the work that we publish. This form provides structure for consistency and transparency in reporting. For further information on Nature Portfolio policies, see our Editorial Policies and the Editorial Policy Checklist.

Statistics

For all statistical analyses, confirm that the following items are present in the figure legend, table legend, main text, or Methods section.

n/a	Confirmed	
☐	☒	The exact sample size (*n*) for each experimental group/condition, given as a discrete number and unit of measurement
☐	☒	A statement on whether measurements were taken from distinct samples or whether the same sample was measured repeatedly
☐	☒	The statistical test(s) used AND whether they are one- or two-sided<br>*Only common tests should be described solely by name; describe more complex techniques in the Methods section.*
☒	☐	A description of all covariates tested
☒	☐	A description of any assumptions or corrections, such as tests of normality and adjustment for multiple comparisons
☐	☒	A full description of the statistical parameters including central tendency (e.g. means) or other basic estimates (e.g. regression coefficient) AND variation (e.g. standard deviation) or associated estimates of uncertainty (e.g. confidence intervals)
☐	☒	For null hypothesis testing, the test statistic (e.g. *F*, *t*, *r*) with confidence intervals, effect sizes, degrees of freedom and *P* value noted<br>*Give P values as exact values whenever suitable.*
☒	☐	For Bayesian analysis, information on the choice of priors and Markov chain Monte Carlo settings
☐	☒	For hierarchical and complex designs, identification of the appropriate level for tests and full reporting of outcomes
☐	☒	Estimates of effect sizes (e.g. Cohen's *d*, Pearson's *r*), indicating how they were calculated

Our web collection on statistics for biologists contains articles on many of the points above.

Software and code

Policy information about availability of computer code

Data collection

-All the wide field microscopy images were captured using a 63x oil immersion objective or 10x objective on a Zeiss Axio Imager M2 microscope fitted with an AxioCam ICc1 digital camera.
-Rescue-STED microscopy was performed on a single-point scanning Expert Line easy3D STED super-resolution microscope (Abberior Instruments GmbH), equipped with a pulsed 775 nm STED depletion laser and two avalanche photodiodes for detection.
-UExM images were acquired on a Leica TCS SP8 microscope.
-SIM images were captured with an inverted microscope using Zeiss Plan-Apochromat 63x/1.4 Oil immersion or Zeiss C-Apochromat 63×/1.2 W Korr M27 water immersion objective on a Zeiss Elyra PS.1 microscope, using the structured illumination microscopy (SIM) technique.
-The tryptic peptides were analysed by liquid chromatography–tandem mass spectrometry.
-Libraries for RNA seq and ChIP seq were sequenced using a NovaSeq 6000 DNA sequencer (Illumina).

Data analysis

-All the wide field microscopy images were analysed with the AxioVision 4.8.2 software.
-Pearson’s colocalization coefficient (R) was calculated using image J software (version 1.44).
-Icy software – version 1.9.10.0. was used to deconvolve the images wherever required.
-STED images were assembled in Fiji (ImageJ-win64) as maximum intensity projections of acquired z-stacks.
-UExM images were analysis was performed using Fiji-Image J and Leica Application Suite X (LAS X) software.
-Processing and export of SIM images and videos were done by Zeiss Zen 2012 Black edition, Service Pack 5 and Zeiss Zen 2.1 Blue edition.
-Mascot (http://www.matrixscience.com/) and MaxQuant (https://www.maxquant.org/) search engines were used for mass spectrometry data analysis.
-PCA was performed using the ClustVis webserver (settings Nipals PCA, no scaling)
-Sequence searches for missing homologues (Mps1, Bub1, Polo, Survivin, Borealin) were performed with HHsearch (Soding, 2005) and the hmmer package (Eddy, 2011).

-FastQC (https://www.bioinformatics.babraham.ac.uk/projects/fastqc/), was used to analyze the raw read quality of RNA seq and ChIP seq libraries. Gene ontology enrichment was done using R package topGO (https://bioconductor.org/packages/release/bioc/html/topGO.html).
- Genome browser tracks for ChIP seq were generated and viewed using the Integrative Genomic Viewer (IGV) with the weight01 algorithm.
- All statistical analyses were performed using GraphPad Prism 9 (GraphPad Software).

For manuscripts utilizing custom algorithms or software that are central to the research but not yet described in published literature, software must be made available to editors and reviewers. We strongly encourage code deposition in a community repository (e.g. GitHub). See the Nature Portfolio guidelines for submitting code & software for further information.

Data

Policy information about availability of data

All manuscripts must include a data availability statement. This statement should provide the following information, where applicable:

- Accession codes, unique identifiers, or web links for publicly available datasets
- A description of any restrictions on data availability
- For clinical datasets or third party data, please ensure that the statement adheres to our policy

The PlasmoDB database was used for protein annotation identified by mass spectrometry for proteomic studies and the data is submitted to PRIDE (PXD043164), Chip seq and RNA seq data are submitted to NCBI (PRJNA808974) , and all raw data are available.

Human research participants

Policy information about studies involving human research participants and Sex and Gender in Research.

Reporting on sex and gender	not applicable
Population characteristics	not applicable
Recruitment	not applicable
Ethics oversight	not applicable

Note that full information on the approval of the study protocol must also be provided in the manuscript.

Field-specific reporting

Please select the one below that is the best fit for your research. If you are not sure, read the appropriate sections before making your selection.

☒ Life sciences ☐ Behavioural & social sciences ☐ Ecological, evolutionary & environmental sciences

For a reference copy of the document with all sections, see nature.com/documents/nr-reporting-summary-flat.pdf

Life sciences study design

All studies must disclose on these points even when the disclosure is negative.

Sample size

The sample sizes are described below. The mice numbers were calculated with the 3Rs (Replacement, refinement and reduction) in mind to produce meaningful, statistically valid and reproducible results as given below, and are based on our extensive experience in this area
3 mice for generation of each transgenic lines (tag lines and knockout/conditional knock down lines)-
10 mice for parasite limiting cloning for each transgenic KO/KD lines (ark2 and EB1)
5 mice for live and fixed cell imaging for each protein at different stages
3 mice for mosquito feeding for every tag lines (ARK2, EB1)
3 mice for generation of each dual tagged lines (ARK2xNDC80;ARK2x kinesin-8B; EB1 x NDC0; EB1 x ARK2; EB1x SAS4)
5 mice /RNA seq and qPCR analysis (ARK2, EB1 and WTGFP)
5 mice / sample for ChIP seq analysis (EB1 and NDC80)
8 mice / sample for immunoprecipitation experiment (EB1, ARK2 and WTGFP)

Data exclusions

Those data are excluded where we needed to optimize the experiments. For example; Immuno-precipitation and mass spectrometry data, Immuno-fluorescence assays

Replication

Most of the experiments were done in triplicate or described in method sections

Randomization

The data analysis was done randomly by different research group without knowing the localization and phenotypic data. e.g. for proteomic studies; cell lysate preparation and immunoprecipitation was done at the University of Nottingham. Tryptic digestion and mass spectrometry analysis was done at the University of Warwick. Preliminary analysis of mass spec data was done again at University of Nottingham. The principle component analysis and phylogeny was done at University of Groningen. None of the researcher was informed the sample details except the gene name.

Blinding	Parasites were genotyped for the gene targeting but this information was not written on the cage cards or accessible to the staff phenotyping the parasite so that parasites are assessed blind. Most of the experiments were performed and analyzed by different groups blindly. For example, widefield microscopy was done at University of Nottingham; UExM was done at University of Geneva; STED microscopy at Ruđer Bošković Institute. ChIP-seq and RNA-seq analysis of KO/KD were done at University of California. Proteomics analysis was done at University of Warwick.

Reporting for specific materials, systems and methods

We require information from authors about some types of materials, experimental systems and methods used in many studies. Here, indicate whether each material, system or method listed is relevant to your study. If you are not sure if a list item applies to your research, read the appropriate section before selecting a response.

Materials & experimental systems

n/a	Involved in the study
☐	☒ Antibodies
☒	☐ Eukaryotic cell lines
☒	☐ Palaeontology and archaeology
☐	☒ Animals and other organisms
☒	☐ Clinical data
☒	☐ Dual use research of concern

Methods

n/a	Involved in the study
☐	☒ ChIP-seq
☒	☐ Flow cytometry
☒	☐ MRI-based neuroimaging

Antibodies

Antibodies used	Anti-GFP-rabbit polyclonal antibody (mAb) (Invitrogen-A1122)<br>Anti-α tubulin mouse mAb (Sigma-T9026)<br>Anti-α-tubulin B-5-1-2, monoclonal, mouse (Sigma-Aldrich, T5168)<br>primary antibodies against α-tubulin and β-tubulin (1:200 dilution, source: AA344 and AA345 from the Geneva antibody facility), anti γ-tubulin antibody (1:500 dilution, source: Sigma T5192) and anti HA antibody (3F10) (1:250 dilution, source: Roche-12158167001).<br>Secondary antibodies were Alexa 488 conjugated anti-mouse IgG (Invitrogen-A11004) and Alexa 568 conjugated anti-rabbit IgG (Invitrogen-A11034)<br>Secondary anti-mouse IgG Alexa Fluor 594 (Abcam, ab150112); RRID: AB_2813898 or STAR ORANGE, goat anti-mouse IgG, (Abberior GmbH, STORANGE-1001-500UG)<br>Secondary antibodies anti-guinea pig Alexa 647, anti-rabbit Alexa 405 and anti-rat Alexa 488 (source: Invitrogen). Atto 594 NHS-ester (Merck 08741).
Validation	All the primary antibodies used in this study have been validated and published.<br>Anti-GFP-rabbit polyclonal antibody (mAb) (Invitrogen-A1122) and Anti-α tubulin mouse mAb (Sigma-T9026) are validated by Zeeshan et al, 2022; PLOS Biology.<br>Anti-α-tubulin B-5-1-2, monoclonal, mouse (Sigma-Aldrich, T5168) primary antibodies against α-tubulin and β-tubulin (1:200 dilution, source: AA344 and AA345 from the Geneva antibody facility), anti γ-tubulin antibody (1:500 dilution, source: Sigma T5192) and anti HA antibody (3F10) (1:250 dilution, source: Roche-12158167001) were validated by Rashpa et al, 2022; PLOS pathogens

Animals and other research organisms

Policy information about studies involving animals; ARRIVE guidelines recommended for reporting animal research, and Sex and Gender in Research

Laboratory animals	Six- to eight-week-old female CD1 outbred mice from Charles River laboratories were used for all experiments. The conditions of mice kept are a 12hour light and 12hour dark (7 till 7) light cycle, the room temperature is kept between 20-24 degrees celcius and the humidity is kept between 40-60%.
Wild animals	NA
Reporting on sex	In this study it was not really important to choose female or male mice because we used them as a media/vehicle to grow Plasmodium and did not study the effect of infection on mice. The important part was to grow the parasite in mice with sustainable parasitaemia that could be easily handled and managed. The Plasmodium grow exactly same way in both sexes, that we have tested previously. We used here female mice without any specific reason but found them less aggressive and easy to handle.
Field-collected samples	No field collected samples were used in the study.
Ethics oversight	The animal work passed an ethical review process and was approved by the United Kingdom Home Office. Work was carried out under UK Home Office Project Licenses (30/3248 and PDD2D5182) in accordance with the UK 'Animals (Scientific Procedures) Act 1986'.

Note that full information on the approval of the study protocol must also be provided in the manuscript.

ChIP-seq

Data deposition

☒ Confirm that both raw and final processed data have been deposited in a public database such as GEO.

☒ Confirm that you have deposited or provided access to graph files (e.g. BED files) for the called peaks.

Data access links<br>*May remain private before publication.*	https://www.ncbi.nlm.nih.gov/search/all/?term=PRJNA808974
Files in database submission	2 replicates of ChIP-seq for EB1-GFP, and ChIP input for EB1-GFP. (Also, RNA-seq files for EB1-KO, ARK2-PTD, and WT gametocytes).
Genome browser session<br>(e.g. UCSC)	Not available

Methodology

Replicates	Two biological replicates for EB1-GFP ChIP-seq, along with input. Genome browser visualization showed high degree of agreement between the two replicates.
Sequencing depth	Sequencing was paired-end, 100 bp. Approximately 10 million pairs for each sample. About 7-8 million uniquely mapped pairs for each sample.
Antibodies	GFP antibody - Abcam ab290 anti-rabbit.
Peak calling parameters	Peak calling was not performed, due to the clarity of the peaks at the centromeres and lack of other peaks.
Data quality	Adapter trimming was done using Trimmomatic and trimming of bases from reads by base quality was done using Sickle, to ensure high-quality reads. Only uniquely mapped, properly paired reads were kept, and likey PCR duplicates were removed, so that only true signal was retained. Peak calling was not performed.
Software	FastQC (https://www.bioinformatics.babraham.ac.uk/projects/fastqc/), was used to analyze raw read quality. Any adapter sequences were removed using Trimmomatic (http://www.usadellab.org/cms/?page=trimmomatic). Bases with Phred quality scores below 25 were trimmed using Sickle (https://github.com/najoshi/sickle). The resulting reads were mapped against the P. berghei ANKA genome (v36) using Bowtie2 (version 2.3.4.1). Using Samtools, only properly paired reads with mapping quality 40 or higher were retained and reads marked as PCR duplicates were removed by PicardTools MarkDuplicates (Broad Institute).
